# The association between maternal social support levels during pregnancy and child development at three years of age: the Japan Environment and Children’s Study

**DOI:** 10.1265/ehpm.23-00211

**Published:** 2024-03-19

**Authors:** Yousuke Imanishi, Satoyo Ikehara, Yuri Aochi, Tomotaka Sobue, Hiroyasu Iso, The Japan Environment and Children’s Study Group

**Affiliations:** 1 Division of Environmental Medicine and Population Sciences, Department of Social and Environmental Medicine, Graduate School of Medicine, Osaka University, Suita City, Osaka, Japan; 2 Osaka Regional Center for Japan Environment and Children’s Study (JECS), Osaka University, Suita City, Osaka, Japan; 3 Institute for Global Health Policy Research, Bureau of International Health Cooperation, National Center for Global Health and Medicine, Tokyo, Japan

**Keywords:** Social support, Pregnancy, Child development, Prospective study

## Abstract

**Background:**

Social relationships are essential in maintaining the physical and mental health of mothers and their children. However, there is limited evidence on how social support provided to the mother during pregnancy could impact child development. Herein, we examined whether maternal social support levels during pregnancy was associated with the risk of developmental delay in 3-year-old children.

**Methods:**

Overall, 68,442 mother-child pairs completed questionnaires on maternal social support during pregnancy and development delay in 3-year-old children. The maternal social support level was evaluated using four items. The risk of development delay was evaluated using the Japanese version of the Ages and Stages Questionnaire-3 (ASQ-3) with five domains of communication, gross motor, fine motor, problem-solving, and personal-social. Odds ratios (OR) and 95% confidence intervals (CI) were calculated using logistic regression according to the quintiles of maternal social support levels after adjusting for potential confounding factors.

**Results:**

Social support during pregnancy was associated with a lower risk of development delay at 3 years of age. Beneficial effects were detected in all domains of the ASQ-3 (p for trend <0.001). Multivariable ORs (95% CIs) for the highest versus lowest quartiles of maternal social support level were 0.57 (0.50–0.65) for communication, 0.49 (0.43–0.55) for gross motor delay, 0.58 (0.53–0.64) for fine motor delay, 0.56 (0.51–0.62) for problem-solving delay, and 0.52 (0.45–0.60) for personal social delay. The associations remained unchanged when stratified by maternal education level, paternal education level, living with children, household income, and postpartum depression.

**Conclusion:**

Maternal social support during pregnancy was inversely associated with the risk of developmental delay at 3 years of age.

## Background

Individual’s social and organized connections, such as family, neighborhood networks, and marital relationships may improve the individuals’ health and quality of life [1]. The presence of trusted acquaintances fostered high social support in the community to raise their children among mothers in post-disaster communities [2].

Pregnancy, inherently accompanied by physical and emotional transformation, places a substantial demand on women’s psychological resilience. Social support such as emotional empathy from families and friends, treated neighborhoods could alleviate the challenges of this transition [3] and also positively influence the in-utero environment through glucocorticoid metabolism [4, 5]. Pregnant women with low social support were at an increased risk for mental health problems, such as depression, anxiety, and self-harm [6]. Social support buffered the association between stressful life events and depression during pregnancy among women [7].

Maternal social support during pregnancy was positively associated with child development at 2 to 3.5 years of age according to previous studies in the United States, Europe, and Canada [8–10]. However, the sample size was limited to approximately 700 to 1,600 [8–10] and potential confounding was not adjusted sufficiently [9, 10]. We therefore attempted to investigate the association between maternal social support during pregnancy and the risk of child development delay using the Japan Environment and Children’s Study (JECS) by assessing approximately 68,442 mother-child pairs.

We hypothesized that high levels of maternal social support during pregnancy would positively affect child development at 3 years of age, independent of maternal age, socioeconomic factors, gestational week, birth weight, and maternal postpartum depression.

## Methods

### Study cohort

The present study used data from the JECS. The JECS is a nationwide birth cohort study funded by the Ministry of the Environment, Japan. A total of 104,062 fetuses were registered across 15 Regional Centers (Hokkaido, Miyagi, Fukushima, Chiba, Kanagawa, Koshin, Toyama, Aichi, Kyoto, Osaka, Hyogo, Tottori, Kochi, Fukuoka, and South Kyushu/Okinawa) between January 2011 and March 2014. This study analyzed the jecs-ta-20190930 dataset, released in October 2019 and revised in November 2022. The JECS protocol was reviewed and approved by the Ministry of the Environment’s Institutional Review Board of Epidemiological Studies and the Ethics Committees of all participating institution. Written informed consent was obtained from all participants. The details of the JECS project have been described elsewhere [11, 12].

We used the data from questionnaires administered to mothers. Pregnant women completed self-reported questionnaires during their first, second and third trimesters. The questionnaire included questions regarding the mothers’ social support, socioeconomic status, medical history, and lifestyle factors. Additionally, mothers answered questionnaires regarding their children at 3 years of age.

Out of the registered 104,062 fetuses, a total of 100,303 live births were recorded. After excluding 1,891 multiple births, 98,412 singleton births remained. We next excluded unidentified and missing data of children’s sex (n = 18) and maternal age (n = 7); infants with eye (n = 60) and ear (n = 615) abnormalities; those with chromosomal abnormalities (n = 145); mother’s with mental disorders (n = 7,909); those with missing data on birth weight (n = 314), maternal social support during pregnancy (n = 3,226), and the Ages and Stages Questionnaire, third edition (ASQ-3) [13] at 3 years of age (n = 17,676).

**Fig. 1:**
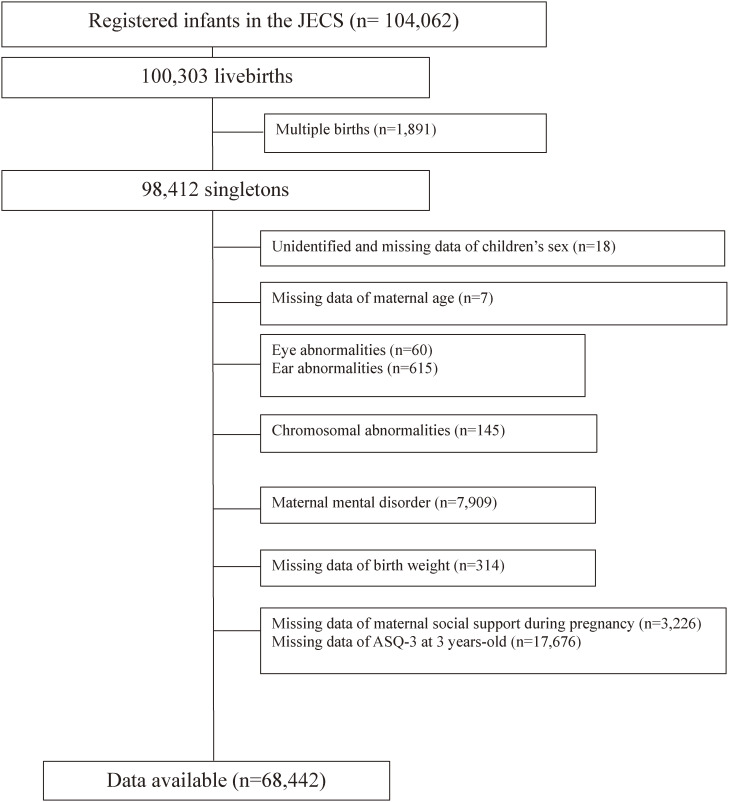
Flow chart of participant selection.

We defined mothers with mental disorders as those suffering from depression, autonomic neuropathy, anxiety disorders, or schizophrenia assessed in the second half of pregnancy and at the childbirth or taking medication for these disorders assessed in the first and second half of pregnancy. Finally, 68,442 mother-child pairs were included in the analysis. (Fig. 1).

### Measurements

#### Exposures

The primary exposure was maternal social support levels during pregnancy, which were evaluated using the following four items: [1] ‘Is there someone available to you who shows you love and affection?’ [2] ‘Is there someone you can count on to provide you with emotional support (talking about problems or helping you make difficult decisions)?’, [3] ‘How often do you have as much contact as you would like with someone you feel close to someone in whom you can trust and confide?’ and [4] ‘Number of friends/neighbors to whom you can talk casually about your concern?’. Questions 1, 2, and 3 were scored using a 5-point scale (0 = not always, 1 = sometimes, 2 = to a certain extent, 3 = almost always, 4 = always), and question 4 was scored using a 3-point scale (0 = none, 1 = 1–2 people, 2 = more than 3 people). Therefore, the total score for social support ranged from 0 to 14. To assess the internal consistency in these indicators to measure child development, we calculated the standardized Cronbach’s alpha (α = 0.74). We used categorical values (quintiles) for maternal social support. These questions were modified from the Social Support Questionnaire [14].

#### Outcomes

The ASQ-3 is composed of 21-age specific questionnaires for children ages 1 to 66 months to evaluate children’s development in 5 domains (communication, gross motor, fine motor, problem-solving, personal-social), with 6 questions result for each domain. Parents respond to these questions describing behavioral skills and abilities; “yes” (10 points), “sometimes” (5 points), or “not yet” (0 points). Parents may skip an item if they are not sure how to answer it or concerned about their poor child’s performance. If one or two scores were missing, the average score of the remaining items was substituted to calculate the overall score [15]. Following the recommended ASQ-3 procedures, modified age was used to determine the ASQ-3 for preterm infants (<37 weeks gestation). The cutoff scores (−2.0 standard deviation (SD)) were 29.95 for communication, 39.26 for gross motor, 27.91 for fine motor, 30.03 for problem-solving, and 26.89 for personal-social. These cutoff scores were based on previously validated cutoff scores for Japanese children [16]. We determined that children whose score was below the cutoff value were at risk for development delay.

### Statistical analyses

We calculated the mean values (SD) and risk factor prevalence according to maternal social support levels during pregnancy. A logistic regression model was used to estimate odds ratios (ORs) and 95% confidence intervals (CIs) for social support quintiles. The lowest social support quintile was used as the reference for the analysis. The multivariable ORs (95% CI) were estimated after adjustment for maternal age (continues) and other covariates, including residential area (15 regional centers), maternal education (junior high school or high school, vocational school, junior college, university or graduate school, or missing), paternal education (junior high school or high school, vocational school, junior college, university or graduate school, or missing), household income (<4 million, ≥4 and <6 million, ≥6 and <8 million, ≥8 and <10 million and ≥10 million yen, or missing), living with other children (yes/no or missing), and living with a partner (yes/no or missing) in model 1, and further adjustment for gestational week (continuous), birth weight (continuous), postpartum depression evaluated by the Edinburgh Postnatal Depression Scale (EPDS score) (<9, ≥9, or missing) added in model 2. The reason for constructing model 2 was to adjust for potential confounding factors that may affect development after birth. Missing data for these confounding factors were included as categorical variables in the model. Tests for trends were conducted using the median value of each category of social support levels. We also used step-wise multiple linear regression to examine which factors other than social support score (continuous) was associated with the child development score (continuous).

All statistical analyses were performed using the SAS software (version 9.4; SAS Institute Inc., Cary, NC, USA).

## Results

Table 1 presents the mean values and proportions of maternal and child characteristics according to quintiles of maternal social support levels during pregnancy. The highest quintile of maternal social support level was less likely to have smoking, living with other children, and postpartum depression and more likely to have education, household income, living with a partner, and partner’s education (p value <0.001 for each).

**Table 1:** Characteristics of mother–child pairs according to maternal social support level during pregnancy

	**Quintiles of maternal social support**				

	**Q1**	**Q2**	**Q3**	**Q4**	**Q5**
N	13919	11051	11502	19373	12597
Maternal age (year ± SD)	31.2 ± 5.2	31.3 ± 5.1	31.5 ± 4.9	31.6 ± 4.7	31.6 ± 4.6
Pre-pregnancy body mass index (kg/m^2^ ± SD)	21.4 ± 3.3	21.2 ± 3.2	21.1 ± 3.2	21.0 ± 3.0	21.0 ± 3.1
Baby’s sex (boy, %)	7193 (51.7)	5564 (50.4)	5860 (51.0)	9984 (51.5)	6507 (51.7)
Birth weight (g ± SD)	3026 ± 405	3026 ± 412	3030 ± 416	3027 ± 403	3033 ± 402
Gestational week (weeks ± SD)	38.8 ± 1.5	38.9 ± 1.5	38.8 ± 1.5	38.9 ± 1.5	38.9 ± 1.5
Maternal smoking during pregnancy, n (%)					
Never	7719 (55.5)	6377 (57.7)	6979 (60.7)	12355 (63.8)	8338 (66.2)
Quit before pregnant	3427 (24.6)	2648 (24.0)	2747 (23.9)	4416 (22.8)	2790 (22.2)
Quit after pregnant	2048 (14.7)	1499 (13.6)	1322 (11.5)	2004 (10.3)	1131 (9.0)
Current smoking	609 (4.4)	452 (4.1)	382 (3.3)	498 (2.6)	274 (2.2)
Missing	116 (0.8)	75 (0.7)	72 (0.6)	100 (0.5)	64 (0.5)
Maternal education, n (%)					
High school or less	5912 (42.5)	4092 (37.0)	3881 (33.7)	5485 (28.3)	3113 (24.7)
Vocational school	3171 (22.8)	2572 (23.3)	2661 (23.1)	4636 (23.9)	2855 (22.6)
Junior college	2360 (17.0)	2137 (19.3)	2311 (20.1)	4107 (21.2)	2877 (22.8)
University or graduate school	2436 (17.5)	2225 (20.1)	2613 (22.7)	5099 (26.3)	3719 (29.5)
Missing	40 (0.3)	25 (0.2)	36 (0.3)	46 (0.2)	33 (0.3)
Paternal education, n (%)					
High school or less	6757 (48.6)	4915 (44.5)	4790 (41.6)	7248 (37.4)	4466 (35.5)
Vocational school	2445 (17.6)	2025 (18.3)	2158 (18.8)	3717 (19.2)	2345 (18.6)
Junior college	626 (4.5)	480 (4.3)	522 (4.5)	865 (4.5)	520 (4.1)
University or graduate school	3939 (28.3)	3547 (32.1)	3948 (34.3)	7329 (38.4)	5206 (41.3)
Missing	152 (1.1)	84 (0.8)	84 (0.7)	114 (0.6)	60 (0.5)
Income, n (%)					
<4 million yen	5881 (42.3)	4258 (38.5)	4111 (35.7)	6311 (32.6)	3933 (31.2)
≥4 and <6 million yen	4167 (29.9)	3420 (31.0)	3723 (32.4)	6349 (32.8)	4127 (32.8)
≥6 and <8 million yen	1835 (13.2)	1623 (14.7)	1753 (15.2)	3264 (16.9)	2288 (18.2)
≥8 and <10 million yen	661 (4.8)	632 (5.7)	745 (6.5)	1445 (7.5)	978 (7.8)
≥10 million yen	413 (3.0)	387 (3.5)	473 (4.1)	948 (4.9)	651 (5.2)
Missing	962 (6.9)	731 (6.6)	697 (6.1)	1056 (5.5)	620 (4.9)
Living with partner, n (%)					
Yes	12753 (91.6)	10206 (92.4)	10761 (93.6)	18241 (94.2)	11806 (93.7)
No	1109 (8.0)	798 (7.2)	695 (6.0)	1071 (5.5)	750 (6.0)
Missing	57 (0.4)	47 (0.4)	46 (0.4)	61 (0.3)	41 (0.3)
Living with other children, n (%)					
Yes	7777 (55.9)	6106 (55.3)	6163 (53.6)	10073 (52.0)	6683 (53.1)
No	6085 (43.7)	4898 (44.3)	5293 (46.0)	9239 (47.7)	5873 (46.6)
Missing	57 (0.4)	47 (0.4)	46 (0.4)	61 (0.3)	41 (0.3)
Postpartum depression, n (%)					
EPDS score <9	11534 (82.9)	9880 (89.4)	10476 (91.1)	18089 (93.4)	11994 (95.2)
EPDS score ≥9	2318 (16.7)	1129 (10.2)	983 (8.6)	1211 (6.3)	567 (4.5)
Missing	67 (0.5)	42 (0.4)	43 (0.4)	73 (0.4)	36 (0.3)

Table 2 shows the ORs (95% CI) for developmental delay in 3-year-old children according to the quintiles of maternal social support level during pregnancy. The higher social support was associated with a lower risk of child developmental delay at 3 years of age. The multivariable OR (95%CI) for the highest versus lowest quantities of maternal social support levels were 0.57 (0.50–0.65) for communication (p for trend <0.001), 0.49 (0.43–0.55) for gross motor delay (p for trend <0.001), 0.58 (0.53–0.64) for fine motor delay (p for trend <0.001), 0.56 (0.51–0.62) for problem-solving delay (p for trend <0.001), and 0.52 (0.45–0.60) for personal social delay (p for trend <0.001).

**Table 2:** Odds ratio for child development delay according to maternal social support level during pregnancy

	**Quintiles of maternal social support**					**p for trend**

	**Q1**	**Q2**	**Q3**	**Q4**	**Q5**	
N	13919	11051	11502	19373	12597	
Communication						
Cases	760	444	434	639	345	
OR (95%CI)	1.00	0.73 (0.64–0.82)	0.68 (0.60–0.77)	0.59 (0.53–0.66)	0.49 (0.43–0.56)	<0.001
Adjusted OR (95%CI) 1	1.00	0.75 (0.66–0.84)	0.71 (0.63–0.80)	0.64 (0.57–0.71)	0.54 (0.47–0.61)	<0.001
Adjusted OR (95%CI) 2	1.00	0.78 (0.69–0.88)	0.75 (0.66–0.85)	0.68 (0.60–0.75)	0.57 (0.50–0.65)	<0.001
Gross motor						
Cases	831	520	497	778	391	
OR (95%CI)	1.00	0.78 (0.69–0.87)	0.70 (0.63–0.79)	0.64 (0.58–0.71)	0.49 (0.44–0.56)	<0.001
Adjusted OR (95%CI) 1	1.00	0.79 (0.71–0.89)	0.72 (0.65–0.81)	0.67 (0.61–0.75)	0.52 (0.46–0.59)	<0.001
Adjusted OR (95%CI) 2	1.00	0.77 (0.69–0.86)	0.70 (0.62–0.78)	0.64 (0.57–0.70)	0.49 (0.43–0.55)	<0.001
Fine motor						
Cases	1395	905	834	1290	690	
OR (95%CI)	1.00	0.80 (0.74–0.87)	0.70 (0.64–0.77)	0.64 (0.59–0.69)	0.52 (0.47–0.57)	<0.001
Adjusted OR (95%CI) 1	1.00	0.82 (0.75–0.89)	0.72 (0.66–0.79)	0.67 (0.62–0.73)	0.56 (0.50–0.61)	<0.001
Adjusted OR (95%CI) 2	1.00	0.84 (0.77–0.92)	0.75 (0.68–0.82)	0.70 (0.65–0.76)	0.58 (0.53–0.64)	<0.001
Problem-solving						
Cases	1377	811	831	1232	654	
OR (95%CI)	1.00	0.72 (0.66–0.79)	0.71 (0.65–0.78)	0.62 (0.57–0.67)	0.50 (0.45–0.55)	<0.001
Adjusted OR (95%CI) 1	1.00	0.74 (0.67–0.81)	0.73 (0.66–0.80)	0.64 (0.59–0.70)	0.53 (0.48–0.58)	<0.001
Adjusted OR (95%CI) 2	1.00	0.76 (0.69–0.83)	0.76 (0.69–0.83)	0.68 (0.62–0.74)	0.56 (0.51–0.62)	<0.001
Personal-social						
Cases	643	381	346	542	265	
OR (95%CI)	1.00	0.74 (0.65–0.84)	0.64 (0.56–0.73)	0.60 (0.53–0.67)	0.44 (0.38–0.51)	<0.001
Adjusted OR (95%CI) 1	1.00	0.76 (0.67–0.86)	0.66 (0.58–0.76)	0.63 (0.56–0.70)	0.48 (0.41–0.55)	<0.001
Adjusted OR (95%CI) 2	1.00	0.79 (0.69–0.90)	0.70 (0.61–0.80)	0.67 (0.60–0.76)	0.52 (0.45–0.60)	<0.001

OR, odds ratio; CI, confidence interval.

Model 1: Adjusted for regional centers, maternal age, maternal education, paternal education, household income, living with children, and living with a partner.

Model 2: Adjusted further for gestational weeks, birth weight, and postpartum depression.

According to the stepwise multiple regression analysis, social support during pregnancy, as well as gestational week, household income were positively associated with child development while maternal age and postpartum depression was inversely associated with it consistently for all of the ASQ-3 domains (Table 3).

**Table 3:** Stepwise multiple linear regression for child development

	**β**	**Std. error**	**P value**
**Communication**			
Social support	0.278	0.014	<0.001
Maternal age	−0.127	0.009	<0.001
Postpartum depression	−1.672	0.144	<0.001
Gestational week	0.428	0.027	<0.001
Maternal education	1.105	0.095	<0.001
Paternal education	0.233	0.089	0.009
Household income	0.533	0.039	<0.001
Living with children	0.136	0.084	0.105

**Gross Motor**			
Social support	0.194	0.011	<0.001
Maternal age	−0.073	0.007	<0.001
Postpartum depression	−1.303	0.114	<0.001
Gestational week	0.378	0.025	<0.001
Birth weight	0.229	0.044	<0.001
Paternal education	−0.169	0.067	0.012
Household income	0.179	0.030	<0.001
Living with children	0.928	0.068	<0.001
Living with a partner	−0.292	0.140	0.036

**Fine motor**			
Social support	0.350	0.016	<0.001
Maternal age	−0.092	0.011	<0.001
Postpartum depression	−2.468	0.175	<0.001
Gestational week	0.584	0.033	<0.001
Maternal education	0.894	0.115	<0.001
Paternal education	0.372	0.108	0.005
Household income	0.479	0.048	<0.001
Living with children	1.979	0.102	<0.001

**Problem-solving**			
Social support	0.285	0.014	<0.001
Maternal age	−0.121	0.009	<0.001
Postpartum depression	−2.038	0.149	<0.001
Birth weight	0.107	0.033	0.063
Gestational week	0.421	0.058	<0.001
Maternal education	0.514	0.098	<0.001
Paternal education	0.239	0.092	0.009
Household income	0.357	0.040	<0.001
Living with children	0.792	0.088	<0.001
Living with a partner	−0.474	0.183	0.010

**Personal-social**			
Social support	0.257	0.013	<0.001
Maternal age	−0.202	0.009	<0.001
Postpartum depression	−2.234	0.139	<0.001
Birth weight	−0.123	0.054	0.021
Gestational week	0.475	0.031	<0.001
Maternal education	0.500	0.091	<0.001
Paternal education	−0.255	0.085	0.003
Household income	0.586	0.037	<0.001
Living with children	1.833	0.081	<0.001

## Discussion

In this large prospective study of approximately 67,000 mother-child pairs, we found that higher social support during pregnancy was positively associated in a dose response fashion with a lower risk of child development delay at 3 years of age, independent of maternal age, socioeconomic factors, gestational week, birth weight, and maternal postpartum depression. Our study extended the findings from the previous smaller studies [8–10] with confirming the robust dose-response relation in all of ASQ-3 domains.

There are multiple possible mechanisms by which the social support during pregnancy has an impact on reducing child development delay. First, the experience of stressful life events such as money, employment problems and moving were associated with depression during pregnancy, but the social support during pregnancy buffer the association [7]. A meta-analysis of 64,449 pregnant from 67 studies showed that low social support was associated with psychological problems such as depression and anxiety during pregnancy [6]. Pregnant women with low social support may have an absence of people to confide in, to obtain important information or advice, and/or to alleviate the negative emotions under distressing situations [17]. Secondly, maternal social support, albeit after birth, was positively associated with psychological well-being and the better language acquisition environment at home for children [18]. We assumed that such a beneficial effect could be obtained by social support during pregnancy, too. Third, it is possible that social support received during pregnancy may improve mother’s psychological stress which can be transmitted to the baby through the placenta, though elevated levels of circulating glucocorticoids under the activated hypothalamic pituitary adrenal axis [4]. Such metabolic alternations may affect fatal brain development directly [3–5].

Our study had several limitations. First, the assessment of child development was based on the caregiver’s self-reports. Although ASQ-3 is a worldwide screening tool, it does not accurately cover all developmental aspects. Assessment of mother’s social-economic status and ACES (Adverse Childhood Experiences study) scores should be assessed at multiple time points as prenatal time and infancy other than 3 years of age. Second, nursery school attendance was not determined. Nursery schools afford children opportunities to interact with their peers, which may positively affect their development in low-income and ethnic minority groups [19].

In conclusion, the higher social support during pregnancy was associated with a lower risk of child development delay at 3 years of age. The further follow-up studies are necessary to elucidate the long-term impact of maternal social support during pregnancy on child development.

## Abbreviations

ASQ-3: Ages and Stages Questionnaire-3
CI: confidence intervals
EPDS: Edinburgh Postnatal Depression Scale
JECS: Japan Environment and Children’s Study
OR: odds ratios
SD: standard deviation

## Declarations

### Availability of data and materials

Data are unsuitable for public deposition due to ethical restrictions and legal framework of Japan. It is prohibited by the Act on the Protection of Personal Information (Act No. 57 of 30 May 2003, amendment on 9 September 2015) to publicly deposit the data containing personal information. Ethical Guidelines for Medical and Health Research Involving Human Subjects enforced by the Japan Ministry of Education, Culture, Sports, Science and Technology and the Ministry of Health, Labour and Welfare also restricts the open sharing of the epidemiologic data. All inquiries about access to data should be sent to: jecs-en@nies.go.jp. The person responsible for handling enquiries sent to this e-mail address is Dr Shoji F. Nakayama, JECS Programme Office, National Institute for Environmental Studies.
